# Rare Report Case of Oral Verruca Vulgaris on Torus Palatinus

**DOI:** 10.1055/s-0041-1732949

**Published:** 2021-10-21

**Authors:** Lee Kian Khoo, Low Eng Chai, Bishwa Prakash Bhattarai, Dinesh Rokaya, Boonaur Yongvanichakorn, Natthamet Wongsirichat

**Affiliations:** 1 Private Practice, Selangor, Malaysia; 2 Sunway Medical Centre, Petaling Jaya, Selangor, Malaysia; 3 Walailak University International College of Dentistry, Walailak University, Bangkok, Thailand

**Keywords:** oral cavity, papilloma, torus, palatal, human papilloma virus

## Abstract

Verruca vulgaris, also known as common warts, is most often seen on the skin of hands and feet. Human papilloma virus (HPV) plays an aetiological role in the development of this lesion. Oral verruca vulgaris (OVV) may occur on the palate, buccal mucosa, and tongue. Although asymptomatic and benign, HPV has been linked to squamous cell carcinoma in the oral cavity and oropharyngeal areas. Therefore, prompt surgical removal of OVV is warranted. We report a case of a OVV in a 48-year-old male patient on palate. The lesion was a white nonscrapable lesion in the middle of a torus palatinus. Excisional biopsy was done together with surgical removal of torus palatinus. Histopathological analysis confirmed the diagnosis of OVV.

## Introduction

### Oral Verruca Vulgaris

Verruca vulgaris is typically seen on hands, feet, toes, and fingers. 1 The occurrence of verruca vulgaris intraorally is less common compared to oral squamous papilloma. 2

Although OVV rarely shows malignant change, it could be transmitted to other sites if left untreated. 3 The mode of transmission of the human papilloma virus (HPV) could be from autoinoculation, oral sex, or vertically from pregnant mother to child. 4 5 6

### Torus Palatinus

Torus palatinus is the bone prominence or the exostosis situated in the median palatine region of the maxilla; it has a high prevalence in Asian and Mongoloid ethnic groups. 7 The lack of vascularity and thin mucosal covering of the tori increases the likelihood of traumatic ulcers and inflammation around the exostosis. 8 The surgical removal of torus palatinus is usually indicated before fitting maxillary dentures or when there is a traumatic ulcer. In some severe instances, ulcers may expose the torus by perforating the mucosa causing dysphagia and halitosis. 9

## Case Report

**Fig. 1:**
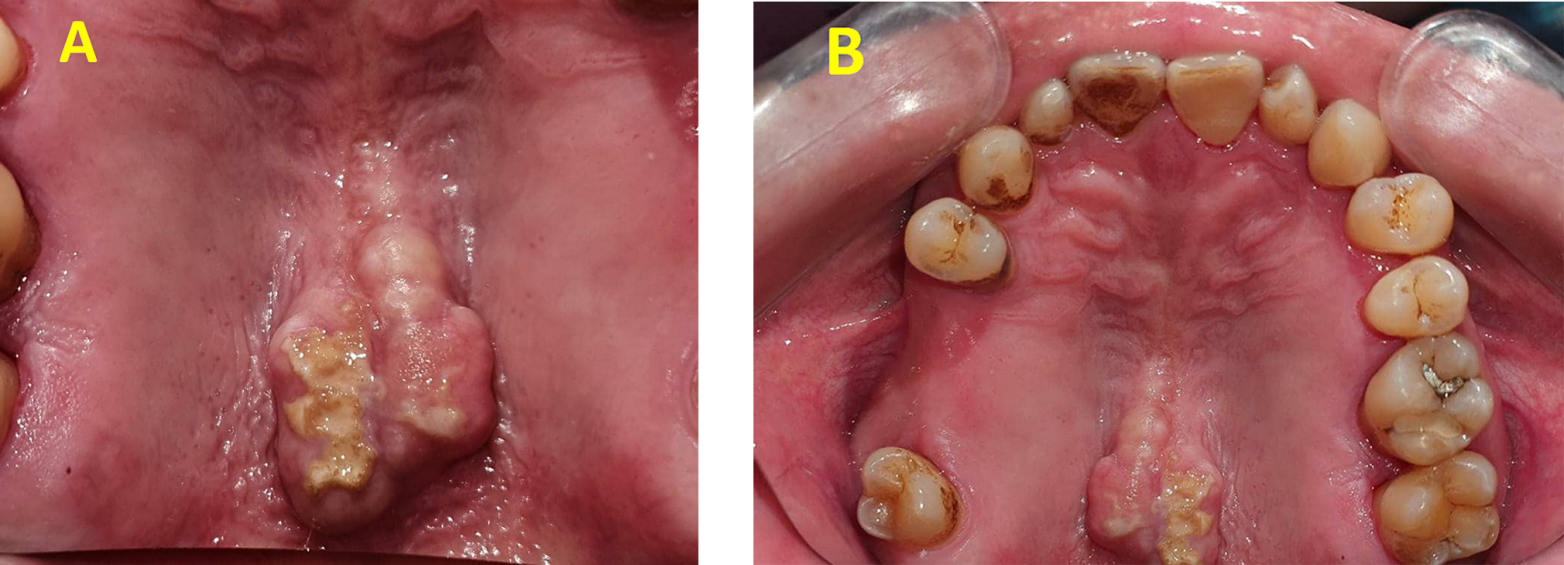
( **A)** Palatal torus with white lesion from the mirror photographic. ( **B** ) Direct photographic of the whole maxillary arch showing moderate sized torus palatinus.

A 48-year-old male patient complained of a whitish growth on his palate. He noticed the lesion approximately 2 months before seeking advice from his dentist. The patient was a smoker averaging 10 cigarettes per day, with occasional alcohol intake. Intraoral examination revealed a whitish lesion confined to the middle of a torus palatinus. The lesion was asymptomatic and was not tender on palpation ( Fig. 1 ).

### Surgical Procedure

**Fig. 2:**
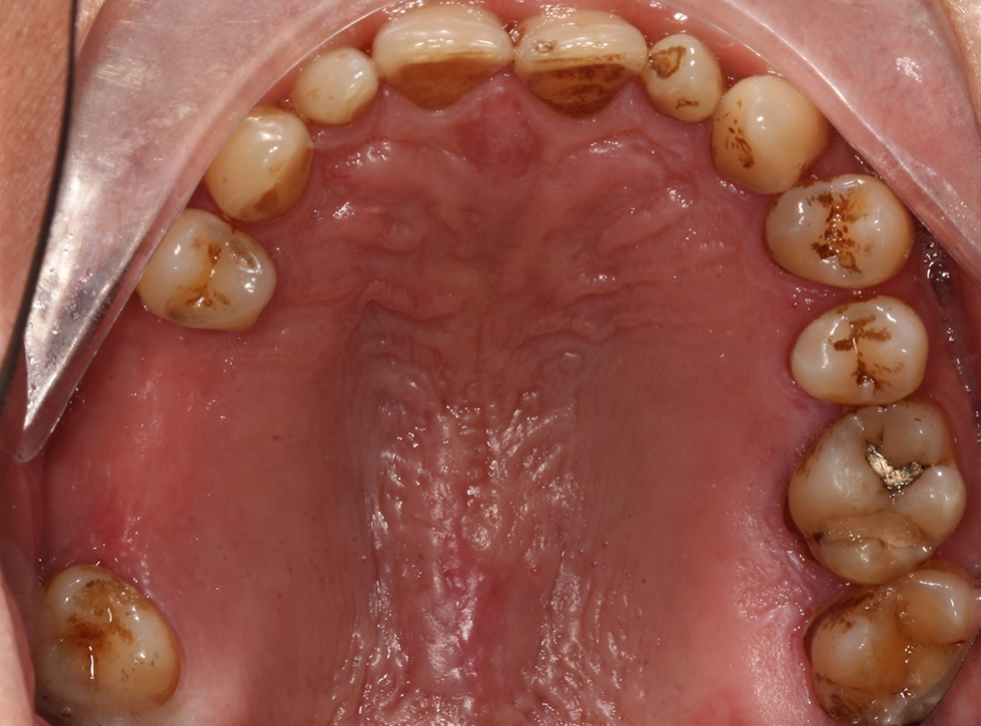
Healing after 14 days postexcisional biopsy and torectomy.

An excisional biopsy was done concomitant with torectomy. The surgical wound was sutured without the use of a splint. Overall 14 days after surgery, the site had healed uneventfully. A follow-up at 6 months revealed no recurrence ( Fig. 2 ). The soft tissue sends to the pathological department for biopsy report.

### Biopsy Result

**Fig. 3:**
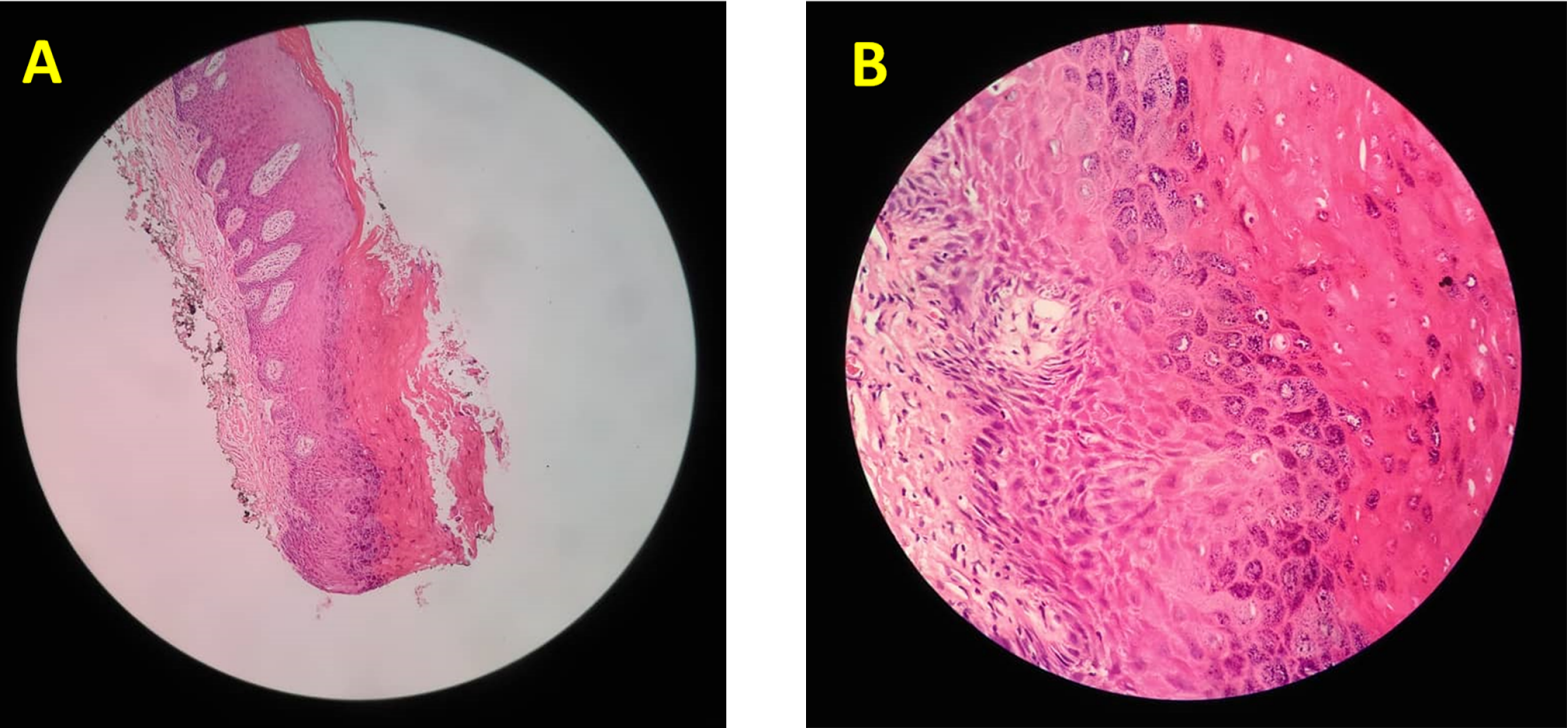
( **A)** Acanthosis with marked hyperkeratosis and bacterial colonies on the surface. ( **B** ) Hypergranulosis is seen with coarse keratohyalin granules.

Fig. 3A and B showed under the microscope; acanthosis is seen with marked hyperkeratosis with bacterial colonies on the surface. Hypergranulosis is seen with coarse keratohyalin granules. Koilocytic change and intracellular bodies are seen. These findings are suggestive of OVV.

## Discussion

The palatal torus is quite common in Southeast Asia; the removal of it is done to help fit dentures that cover the palatal region to prevent discomfort. The thin mucosa covering the palatal torus makes it prone to get ulcerations, which could expose the palatal mucosa to pathogens. 9 The constant trauma to epithelium could potentially be the source of entry of HPV into the basal keratinocytes.

Nonetheless, the histopathological diagnosis of verruca vulgaris is distinctive and enough to differentiate this lesion from other white mucosal lesions in the oral cavity such as frictional keratosis. The presence of intracellular bodies and hypergranulosis, which we see in our case report is not usually seen in frictional keratosis or traumatic keratosis according to Abidullah et al 10 and Sudhakar et al. 11

In this case, the lesion was hyperkeratotic and nonscrapable, which ruled out the presence of candida infection. To the best of this author’s knowledge and based on a thorough online search of previously published articles on OVV, we are the first to document OVV on a palatal torus. This finding is however not entirely surprising because palatal tori are very easily traumatized

The strain of HPV involved in the pathogenesis of OVV is usually HPV type 2 and HPV type 4. 3 These have a low potential for malignant change. 12 Nonetheless, to ascertain the specific type of HPV infection, a polymerase chain reaction assay is necessary. Genital warts or condyloma acuminatum have been shown to carry multiple strains of HPV (both low and high-risk types). 13 With the rising trend of oral sexual practice, reports of HPV infection from genital to oral regions are expected to rise accordingly. 14

Although rare, Atullah et al documented a case of OVV on the lip which transformed into oral squamous cell carcinoma in 4 years when left untreated. 15 Verruca vulgaris on extraoral regions such as the eyelid have also been reported with malignant change into a combination of squamous cell carcinoma and basal cell carcinoma. 16

The benign nature of OVV should not be taken for granted, prompt excisional biopsies are warranted to prevent any potential for malignant change. In this case, concomitant torectomy was done to reduce recurrence, this is based on the belief that trauma to the torus was how HPV inoculated the overlying palatal mucosa.

## References

[1] Mattoo A Bhatia M Verruca vulgaris of the buccal mucosa: a case report J Cancer Res Ther 2018 14 02 454 456 29516939 10.4103/jcrt.JCRT_47_17

[2] Sivapathasundharam B Shifa S Oral verruca vulgaris: report of a rare case Indian J Dent Res 2004 15 01 32 34 15682794

[3] Ural A Arslan S Ersoz Ş Değer B Verruca vulgaris of the tongue: a case report with a literature review Bosn J Basic Med Sci 2014 14 03 136 138 25172971 10.17305/bjbms.2014.3.29 PMC4333997

[4] Bharti A H Chotaliya K Marfatia Y S An update on oral human papillomavirus infection Indian J Sex Transm Dis AIDS 2013 34 02 77 82 24339456 10.4103/2589-0557.120533 PMC3841675

[5] Rautava J Syrjänen S Human papillomavirus infections in the oral mucosa J Am Dent Assoc 2011 142 08 905 914 21804057 10.14219/jada.archive.2011.0297

[6] Toledano-Serrabona J López-Ramírez M Sánchez-Torres A España-Tost A Gay-Escoda C Recurrence rate of oral squamous cell papilloma after excision with surgical scalpel or laser therapy: a retrospective cohort study Med Oral Patol Oral Cir Bucal 2019 24 04 e433 e437 31232385 10.4317/medoral.22943 PMC6667011

[7] Noor M I Tajuddin M F Alam M K Basri R Purmal K Rahman S A Torus palatinus and torus mandibularis in a Malaysian population Int Med J 2013 20 767 769

[8] Bouchet J Hervé G Lescaille G Descroix V Guyon A Palatal torus: etiology, clinical aspect, and therapeutic strategy J Oral Med Oral Surg 2019 25 18

[9] Sinisterra G Álvarez J Molano P E Exposición espontánea de un torus palatino de la línea media[Spontaneous exposition of a midline palatal torus]Biomedica 2013 33 01 31 35 10.1590/S0120-41572013000100004 23715304

[10] Abidullah M Raghunath V Karpe T Clinicopathologic correlation of white, non scrapable oral mucosal surface lesions: a study of 100 cases J Clin Diagn Res 2016 10 02 ZC38 ZC41 10.7860/JCDR/2016/16950.7226 PMC4800649 27042583

[11] Sudhakar S Praveen Kumar B Prabhat M P Prevalence of oral mucosal changes in Eluru, Andhra Pradesh (India) - an institutional study J Oral Health Community Dent 2011 5 42 46

[12] Alvarado J MP Rodríguez V PR Carrasco M FL Ramos V R Carrasco J CR Squamous papilloma in the oral cavity: case presentation and review of the literature J Dent Health Oral Disord Ther 2018 9 04 257 260

[13] Brown D R Schroeder J M Bryan J T Stoler M H Fife K H Detection of multiple human papillomavirus types in Condylomata acuminata lesions from otherwise healthy and immunosuppressed patients J Clin Microbiol 1999 37 10 3316 3322 10488198 10.1128/jcm.37.10.3316-3322.1999 PMC85555

[14] Dos Reis H L Rabelo P C de Santana M R Ferreira D C Filho A C Oral squamous papilloma and condyloma acuminatum as manifestations of buccal-genital infection by human papillomavirus Indian J Sex Transm Dis AIDS 2009 30 01 40 42 21938114 10.4103/2589-0557.55484 PMC3168039

[15] Atullah J K Murad N Khitab U Oral verruca vulgaris: a rare case with transformation of papilloma to well differentiated oral squamous cell carcinoma Pak Oral Dent J 2021 30 327 329

[16] Lai K K Chan E Ko S C Combination of squamous cell carcinoma and basal cell carcinoma arising from a giant verruca vulgaris involving the eyelid Am J Ophthalmol Case Rep 2020 21 100858 33553803 10.1016/j.ajoc.2020.100858 PMC7844115

